# Prevalence, Characterization and Genetic Diversity of *Listeria monocytogenes* in Ready-to-Eat Raw Salmon (*Salmo salar*) and Trout (*Oncorhynchus mykiss*) Products

**DOI:** 10.3390/foods15020385

**Published:** 2026-01-21

**Authors:** Yujie Gong, Lin Yao, Meng Qu, Fengling Li, Yingying Guo, Na Li, Wenjia Zhu, Lianzhu Wang, Peng Wang, Yanhua Jiang

**Affiliations:** 1 Key Laboratory of Testing and Evaluation for Aquatic Product Safety and Quality, Ministry of Agriculture and Rural Affairs, Yellow Sea Fisheries Research Institute, Chinese Academy of Fishery Sciences, No. 106 Nanjing Road, Qingdao 266071, China; 2 College of Food Science and Engineering, Ocean University of China, No. 1299 Sansha Road, West Coast New District, Qingdao 266404, China

**Keywords:** *Listeria monocytogenes*, virulence, antimicrobial resistance, whole genome sequencing, genotyping

## Abstract

*Listeria monocytogenes* is a high-risk pathogenic bacterium associated with ready-to-eat foods and poses a potential threat to consumer health. This study aimed to investigate the prevalence, characterization and genetic diversity of *L. monocytogenes* in ready-to-eat raw salmon and trout products obtained from physical stores and online stores in China. Out of 150 samples analyzed, 23 (15.3%) were positive for *L. monocytogenes*. Among these positive samples, three (12%) were from Japanese restaurants, four (16%) from farmers markets, one (2.9%) from large supermarkets and fifteen (30%) from e-commerce platforms, and only one sample showed a contamination level exceeding 100 most probable number (MPN)/g. The isolates from positive samples demonstrated a concrete public health risk through several findings: twenty-three *L. monocytogenes* exhibited varying degrees of cytotoxicity, ranging from 7.6% to 71.8%. Compared with the reference strain ATCC 19115, five of these isolates were highly cytotoxic, a result that was validated by mouse survival rate experiment, which also confirmed their high virulence at tested dose. All isolates were resistant to cefuroxime sodium, ceftriaxone, cefepime and nalidixic acid, and 13% showed resistance to sulphamethoxazole-trimethoprim. Three serogroups were identified, with serogroup Ⅰ.1 (1/2a, 3a) being the most prevalent (65.2%). These isolates were grouped into eight sequence types, with ST8 (34.8%) and ST87 (30.4%) dominating. All isolates carried virulence genes associated with LIPI-1 andmultiple internalin genes (*inlA*, *inlB*, *inlJ* and *inlK*), confirming their potential pathogenicity. Additionally, the isolates harbored antimicrobial resistance genes *lin* and *FosX*. The five highly virulent isolates exhibited the highest genetic similarity to J2-031 (GCA_000438645.1) and C1-387 (GCA_000438605.1). The results provided valuable information for Chinese regulatory authorities to strengthen the risk monitoring of *L. monocytogenes* in ready-to-eat raw salmon and trout products.

## 1. Introduction

*Listeria monocytogenes* is a significant foodborne pathogen responsible for zoonotic diseases [1,2], which is considered one of the most important contaminants in food processing and ready-to-eat foods [3]. *L. monocytogenes* exhibits high heterogeneity in virulence [4,5]. As an intracellular pathogen, it can be transmitted to other tissues and organs via the intestinal barrier, blood–brain barrier, placental barrier, lymphatic circulation and blood circulation of humans [6]. Infection by *L. monocytogenes* can cause listeriosis, a serious disease primarily affecting pregnant women, newborns, the elderly and other immunocompromised individuals [7]. Listeriosis can lead to severe outcomes such as meningitis, septicemia and spontaneous abortion in pregnant women, with a high mortality rate of 20–30% [8]. It was estimated that approximately 1600 people develop listeriosis annually, resulting in 260 fatalities in the United States [9]. Foods commonly associated with *L. monocytogenes* outbreaks include unpasteurized dairy products, vegetables, deli meat products and raw seafood [10,11,12]. Sashimi, a type of ready-to-eat raw seafood, is popular worldwide due to its unique taste and nutritional benefits. Although sashimi is transported and stored at low temperatures, *L. monocytogenes* is still a threat in these foods since *L. monocytogenes* readily survives and may actually grow under these conditions [13]. The most common fish used for sashimi is salmon, particularly Atlantic salmon (*Salmo salar*). The Chinese market has shown a huge demand for salmon, with annual consumption exceeding 100,000 metric tons [14]. In recent years, rainbow trout (*Oncorhynchus mykiss*) has also become a popular choice for sashimi in certain Chinese market. However, eating sashimi carries a risk of infection with pathogenic microorganisms, such as *Escherichia coli*, *Salmonella*, *Vibrio* and *L. monocytogenes* [15,16]. Since 2015, there were 12 listeriosis outbreaks caused by ready-to-eat salmon products in Denmark, Germany and France [17]. In addition, *L. monocytogenes* isolated from salmon has been associated with no fewer than two known outbreaks of listeriosis in Norway [18]. Thus, *L. monocytogenes* is known as the highest-risk pathogenic microorganism in sashimi.

Quantitative analysis plays a crucial role in the microbiological risk assessment of food products. Although the occurrence of *L. monocytogenes* in salmon products has been reported in previous studies, there are few studies analyzing the quantitative contamination level of *L. monocytogenes* in retail raw salmon and trout products. Traditional methods for analyzing genetic diversity, such as multiple locus variable-number tandem repeat analysis (MLVA) or pulsed-field gel electrophoresis (PFGE), have been widely employed [1,19]. However, these conventional methods are not only laborious but also offer limited resolution for microbial subtyping. Nowadays, whole-genome sequencing (WGS) technology has become a powerful tool to investigate *L. monocytogenes* outbreaks due to its high efficiency, accuracy and comprehensive genomic data [20]. As WGS uses the entire genome of bacteria, it has been applied to reveal the genetic differences among different sequence types, acquisition and evolution in virulence and detect antimicrobial resistance genes as well as virulence genes in *L. monocytogenes* [4,21]. However, studies employing WGS to analyze *L. monocytogenes* isolates from raw salmon and trout products remain scarce.

Currently, the market for raw salmon and trout products in China is gradually expanding. Once an outbreak of listeriosis due to consumption of contaminated raw salmon and trout products occurs, it will lead to huge economic losses, adverse social impacts and disruptions to industrial development. To address this risk, this study investigated the prevalence, characteristics and genetic diversity of *L. monocytogenes* in retail raw salmon (*S. salar*) and trout (*O. mykiss*) products by analyzing the contamination level, phenotypic profiling and WGS, which is of great significance for the prevention and control of listeriosis and enhancing the food safety of retail raw salmon and trout products.

## 2. Materials and Methods

### 2.1. Samples

A total of 150 retail ready-to-eat raw salmon and trout product samples were collected, among which 100 raw salmon (*S. salar*) products samples were randomly sampled from supermarkets, farmers’ markets, so-called “exclusive stores” (defined here as retail outlets specializing in high-end or imported seafood products) and Japanese restaurants located in Qingdao, China; 25 raw salmon (*S. salar*) products and 25 trout (*O. mykiss*) products were from online stores located in different provinces with top 10 sales volumes on e-commerce platforms. Sample size was determined to ensure statistical representativeness, reliable estimation of *L. monocytogenes* contamination in commercial samples and feasibility within available resources while providing adequate data for further analysis. The samples, typically sold in the form of filets or portions, were transported to the laboratory under controlled low-temperature conditions to maintain microbial loads. Testing was conducted immediately upon arrival.

### 2.2. Detection, Enumeration and Isolation of L. monocytogenes in Retail Raw Salmon and Trout Products

The detection, enumeration and isolation of *L. monocytogenes* were carried out according to the method described by Chinese National Standards (GB 4789.30-2016) [22]. For detection of *L. monocytogenes*, 25 g of each sample were fully homogenized in 225 mL primary enrichment medium (*Listeria* enrichment broth base, LB Base) (HuanKai Microbial, Guangzhou, China) containing 0.005 g nalidixonic acid and 0.003 g acriflavine hydrochloride, LB1. After incubation for 24 h at 30 °C, 0.1 mL of primary enrichment was transferred to a tube containing 10 mL of secondary enrichment medium (200 mL LB Base containing 0.004 g nalidixonic acid and 0.005 g acriflavine hydrochloride, LB2). Following incubation at 30 °C for 24 h, a loopful of LB2 enrichment was streaked on the surface of *L. chromogenic* agar plate (CHROMagar, Paris, France) and PALCAM agar plate (Land Bridge, Beijing, China), then incubated at 36 °C for 24 h. Presumptive colonies were subcultured on tryptone soya yeast extract agar (TSYEA) for further identification. The isolates were confirmed to be *L. monocytogenes* by Gram staining, catalase reaction, motility test, hemolysis test and biochemical identification using the commercial kit (Beijing Land Bridge Technology Co., Ltd.). All confirmed *L. monocytogenes* isolates were stored at −80 °C. Prior to phenotypic testing, all isolates were revived from a single freeze–thaw cycle and freshly subcultured.

The enumeration of *L. monocytogenes* was performed using the most probable number (MPN) technique. Briefly, 25 g of each sample in 225 mL LB Base were fully homogenized to make the initial suspension (10^−1^). Then, serial 10-fold dilutions (10^−2^, 10^−3^) were performed. Ten microliters of initial suspension were transferred to each of three tubes containing double-strength LB1, and 1 mL of subsequent dilutions (10^−1^, 10^−2^, 10^−3^) was transferred into each of the three tubes containing 10 mL of single-strength LB1. After incubation for 24 h at 30 °C, 0.1 mL of each tube of LB1 enrichment was transferred to a tube of 10 mL of LB2 and incubated at 30 °C for 24 h. The plating and confirmation of typical colonies were conducted as described above. The results were determined by means of the MPN table provided in GB 4789.30-2016 according to the number of tubes of double- and single-strength medium whose subcultures contain positive *L. monocytogenes*. The minimum detection limit of this technique is 0.3 MPN/g.

### 2.3. Virulence Assays of L. monocytogenes Isolates

#### 2.3.1. Cytotoxicity Assay on Human Colon Carcinoma Caco-2 Cells

The cytotoxicity of *L. monocytogenes* isolates was assessed by measuring the lactate dehydrogenase (LDH) released from infected Caco-2 cells (Pricella Life Science & Technology Co., Ltd., Wuhan, China), following a previously described method [23,24] with minor modifications. Caco-2 cells were cultured in minimum essential medium (MEM) with 20% fetal bovine serum and 1% penicillin–streptomycin solution (Pricella Life Science & Technology Co., Ltd.) at 37 °C with 5% CO_2_ in the cell culture incubator (Heal Force, Shanghai, China). The well-grown Caco-2 cells were seeded with approximately 2 × 10^5^ cells/well in 12-well plates for overnight culture. Additionally, bacterial cultures were grown in brain heart infusion (BHI) broth (Land Bridge Technology, Beijing, China) by shaking culture for 18–24 h. The muddy BHI was adjusted to the required concentration with Dulbecco’s modified eagle medium (DMEM) (Procell, Wuhan, China). And the cell culture medium in the 12-well plates was sucked out and washed twice with phosphate-buffered saline (PBS). Then, Caco-2 cells were infected with *L. monocytogenes* suspended in Dulbecco’s modified eagle medium (DMEM) (Procell, Wuhan, China) (Multiplicity of infection, MOI = 100) for 6 h. The MOI and infection duration were optimized in preliminary experiments to enable comparable assessment of cytotoxicity. Cells treated with DMEM served as negative control, and cells treated with 1% Triton X-100 were positive control. After infection, the cultures were centrifuged at 8000× *g* for 10 min, and the supernatant was used to measure LDH activity by LDH activity assay kit (Sangon Biotech, Shanghai, China). The cytotoxicity of 23 isolates was calculated using the following equation.

Cytotoxicity (%) = (Experimental value − Negative control)/(Positive control − Negative control) × 100(1)

#### 2.3.2. Evaluation of Virulence in a Mouse Model

The virulence of the five strains, characterized by high cytotoxicity, and the four strains with low cytotoxicity was evaluated using mouse mortality assays. The assays were performed according to a previously described method [25] with some modifications. Specific pathogen-free (SPF) female ICR (Institute of Cancer Research) mice, weighing 18–22 g, were purchased from Vital River Laboratory Animal Technology Co., Ltd., Beijing, China. All animal experiments were conducted in accordance with the regulations and norms governing the management of animal experiments and approved for operation by the Animal Care and Use Committee of the Yellow Sea Fisheries Research Institute. The mice were acclimatized under laboratory condition for 7 days prior to the experiment with access to a standard diet and water ad libitum. They were maintained under standard conditions of temperature (20–25 °C) and humidity (40–50%RH) with an alternating 12 h light/dark cycle. A total of 88 mice was randomly divided into 11 groups, with 8 mice in each group. Then, the mice were intraperitoneally injected with 200 μL of bacterial suspension at a dose of 2 × 10^6^ CFU per mouse. The reference strain *L. monocytogenes* ATCC 19115 was used as a high-virulence positive control, and the sterile physiological saline served as the blank control. Following injection, the mice were monitored continuously for 10 days, and survival rates were recorded.

### 2.4. Antimicrobial Susceptibility Testing

Antimicrobial susceptibility testing of *L. monocytogenes* was determined by the disk diffusion method as recommended by the Clinical Laboratory Standards Institute (CLSI) [26], and European Committee on Antimicrobial Susceptibility Testing (EUCAST) [27]. The following antimicrobials disks from Liofilchem (Roseto degli Abruzzi, Italy) were tested: ampicillin (AMP, 10 μg), amoxycillin (AMC, 20 μg), cefazolin (KZ, 30 μg), cefuroxime sodium (CXM, 30 μg), ceftriaxone (CRO, 30 μg), cefepime (FEP, 30 μg), imipenem (IPM, 10 μg), meropenem (MEM, 10 μg), streptomycin (S, 10 μg), amikacin (AK, 30 μg), gentamicin (CN, 10 μg), kanamycin (K, 30 μg), nalidixicacid (NA, 30 μg), ciprofloxacin (CIP, 5 μg), ofloxacin (OFX, 5 μg), tetracycline (TE, 30 μg), doxycycline (DO, 10 μg), sulphamethoxazole-trimethoprim (SXT, 25 µg), chloramphenicol (C, 30 μg), florfenicol (FFC, 30 μg), nitrofurantoin (F, 300 μg), vancomycin (VA, 30 µg), rifampicin (RD, 5 μg). The bacterial cultures were adjusted to 1.0 McFarland turbidity, and 200 μL of the cultures was spread on the Mueller–Hinton agar (Hope Bio, Qingdao, China) plates. The antimicrobials disks were attached on the surface of the plates and incubated at 36 °C for 24 h. After incubation, the diameter of inhibition zone was measured by the vernier caliper. In cases where the CLSI documents and EUCAST guidelines did not provide resistance criteria for *Listeria*, any missing breakpoints were supplemented with those recommended for *Staphylococcus aureus* and *Enterococcus* spp. according to CLSI standards. *S. aureus* ATCC 25923 and *L. monocytogenes* ATCC 19115 were employed as quality control strains [25].

### 2.5. PCR-Serogroup Identification

Five serogroups (I.1: serotypes 1/2a, 3a; I.2: 1/2c, 3c; II.1: 4b, 4d, 4e; II.2: 1/2b, 3b, 7 and III: 4a, 4c) of *L. monocytogenes* isolates were identified by multiple PCR method based on five primers: lmo0737, lmo1118, ORF2819, ORF2110 and prs [28]. The PCR amplification was performed in a final volume of 20 μL that consisted of 0.25 μL (10 μmol/L) of each primer, 2 μL (about 50 ng) of DNA template, 5.5 μL of sterile distilled water and 10 μL of premix *Taq*^TM^ (Takara, Dalian, China). Amplification was conducted under the following condition: initial denaturation step at 94 °C for 5 min, followed by 35 cycles of denaturation at 94 °C for 60 s, annealing at 53 °C for 60 s and extension at 72 °C for 60 s, and an additional extension at 72 °C for 10 min. *L. monocytogenes* ATCC19115 (serotype 4b) was used as control. The PCR amplicons were electrophoresed on an agarose gel and imaged using a Gel Imaging and Analysis System (Vilber Lourmat, Paris, France).

### 2.6. WGS and Genetic Diversity Analysis

#### 2.6.1. Genomic DNA Extraction, Sequencing and Annotation

The genomic DNA of *L. monocytogenes* isolates were extracted using NucleoBond^®^ HMW DNA kit (Macherey-Nagel, Düren, Germany). The concentration of DNA was determined by Qubit 4.0 fluorometer (Thermo, Waltham, MA, USA), and its integrity was assessed by 1% agarose gel electrophoresis. WGS was then performed on 23 *L. monocytogenes strains*, with complete genome sequencing for five high-virulence strains LMSR02, LMSR09, LMSR12, LMSR21 and LMSR22 and draft genome sequencing for the remainder. All sequencing services were provided by Sangon Biotech (Shanghai, China). The draft genome sequencing was conducted on the Illumina NovaSeq 6000 platform (Illumina, San Diego, CA, USA). The genome DNA was randomly fragmented to segments averaging 200–400 bp in size. These fragments then underwent end-repair, 3′ adenylated, adapter ligation and PCR amplifying. After purification using magnetic beads, the library was qualified by the Qubit 4.0 fluorometer (Thermo, Waltham, MA, USA), and its fragment size distribution was assessed by the 2% agarose gel electrophoresis. Raw sequencing reads were filtered via Trimmomatic (v0.36) to remove adaptors and low-quality bases, yielding clean reads. Genome assembly was carried out using SPAdes (v3.15), and gaps were filled with Gapfiller (v1.11). For the complete genome sequencing, a combination of Illumina and PacBio technologies was employed. The genomic DNA was divided into two portions. One portion was randomly fragmented to construct a library with an insert size of 300 bp, which was then sequenced on the Illumina NovaSeq 6000 platform (Illumina, San Diego, CA, USA) using a paired-end 150 bp strategy. The other portion was sheared using g-TUBEs (Covaris, Watertown, MA, USA) according to the target fragment size range. The desired fragments were enriched and purified by AMPure PB beads, and their size distribution was verified by 0.7% agarose gel electrophoresis. Sequencing libraries were prepared from qualified DNA using the SMRTbell^TM^ Template Prep Kit 2.0 (PacBio, Menlo Park, CA, USA). The Sequel II Binding Kit 2.0 was used to mix the DNA library, sequencing primer and polymerase in a certain proportion, after which the library was loaded to initiate real-time single-molecule sequencing on the PacBio Sequel II system. The raw reads were filtered and assembled using Canu with default parameters. The genomic sequences were polished using nextpolish (v1.4.1) and Pilon (v1.18). Gene prediction and functional annotation were performed with the Prokka (v1.10) and the National Center for Biotechnology Information (NCBI) database.

#### 2.6.2. Multi-locus Sequence Typing (MLST) of *L. monocytogenes* Isolates

The MLST of 23 *L. monocytogenes* isolates was performed by analyzing the sequences of the following seven housekeeping genes: *acbZ*, *bglA*, *cat*, *dapE*, *dat*, *ldh* and *lhkA* [29]. The sequence type (ST), clonal complex (CC) and evolutionary lineage were determined by using the BIGSdb-Lm database (http://bigsdb.pasteur.fr/listeria) (accessed on 13 January 2025).

#### 2.6.3. Virulence Gene Analysis

The predicted proteins of all isolates were screened for *L. monocytogenes* virulence genes by performing a BLAST+ (v2.16.0) search against the core dataset of the Virulence Factor Database (VFDB) (pident ≥ 80%, evalue ≤ 1 × 10^−10^) [30] (http://www.mgc.ac.cn/VFs/) (accessed on 16 January 2025).

#### 2.6.4. Antimicrobial Resistance Gene Analysis

The “RGI main” tool (v6.0.3) of the comprehensive antibiotic resistance database (CARD) [31] was used to identify antimicrobial resistance genes in the genomes of the 23 *L. monocytogenes* isolates, with “strict” and “perfect” as the screening criteria (https://card.mcmaster.ca/) (accessed on 20 January 2025).

#### 2.6.5. Single Nucleotide Polymorphisms (SNP)-Based Phylogenetic Analysis

SNP analysis was performed on 23 isolates and 15 *L. monocytogenes* from different regions and years obtained from NCBI. SNPs were identified using Snippy (v4.6.0), with the raw reads of each genome aligned against a reference genome (C1-387: GCA_000438605.1). This reference was selected as it is a complete genome and exhibits high homology to the isolates in this study. A phylogenetic tree was constructed based on SNP mutation information using the neighbor-joining approach in FastTree (v2.1.7). The 15 publicly available comparator genomes obtained from NCBI included the following: Clip80459 (GCA_000026705.1), M7 (GCA_000218305.1), 07PF0776 (GCA_000258905.1), LL195 (GCA_000318055.1), FDAARGOS_57 (GCA_001188045.2), J2-031 (GCA_000438645.1), C1-387 (GCA_000438605.1), J2-064 (GCA_00043865.1), R2-502 (GCA_000438585.1), NE_dc2014 (GCA_000600015.1), 21-03201 (GCA_028673915.1), 19-07394 (GCA_028674095.1), B-33116 (GCA_028743575.1), UKVDL9 (GCA_015689035.1) and NTSN (GCA_000800335.1).

#### 2.6.6. Whole-Genome Average Nucleotide Identity (ANI) and Collinearity Analysis

The ANI analysis was performed on five high-virulence isolates (LMSR02, LMSR09, LMSR12, LMSR21, and LMSR22) against 15 *L. monocytogenes* strains from diverse regions and years obtained from NCBI. Genome collinearity analysis was conducted using Mauve software (v2.4.0).

### 2.7. Statistical Analysis

Statistical analyses were performed using one-way analysis of variance (ANOVA) with Dunnett’s multiple comparisons test (GraphPad Prism, v9.0) to compare the cytotoxicity on Caco-2 cells of 23 *L. monocytogenes* isolates and ATCC19115. The survival of mice following injection with the isolates and ATCC19115 were compared using Kaplan–Meier survival analysis.

## 3. Results

### 3.1. Contamination Level of L. monocytogenes in Retail Raw Salmon–Trout Products

*L. monocytogenes* was detected in 15.3% (23/150) of the analyzed raw salmon and trout samples (Table 1). Of the total samples, 127 were determined to be negative for *L. monocytogenes*, with contamination levels below the detection limit of 0.3 MPN/g. The positive samples exhibited a wide range of contamination levels varying from 0.36 to 1100 MPN/g. Notably, *L. monocytogenes* was not detected in any of the 15 samples collected from the exclusive stores. In contrast, *L. monocytogenes* was present in 12% (3/25) of samples from Japanese restaurants and 16% (4/25) from farmers’ markets, with the contamination level ranging from 2.3 to 43 MPN/g and 0.92 to 9.2 MPN/g, respectively. Large supermarkets demonstrated the lowest prevalence, with only one out of 35 (2.9%) samples testing positive, showing a minimal contamination level of 0.36 MPN/g. What was of particular concern was the contamination observed in samples from e-commerce platforms, which showed the highest prevalence of 30% (15/50), with the most severe contamination level peaking at 1100 MPN/g. In the present study, this one sample from an e-commerce platform exceeded the limit of *L. monocytogenes* set by the Chinese National Standard GB 29921-2021 [32] (100 CFU/g for ready-to-eat raw animal aquatic products)—under the assumption that MPN/g is equivalent to CFU/g. A single representative isolate from each of the 23 positive samples was selected for subsequent analysis.

### 3.2. Virulence of L. monocytogenes Isolates

#### 3.2.1. Cytotoxicity in Caco-2 Cells

**Figure 1:**
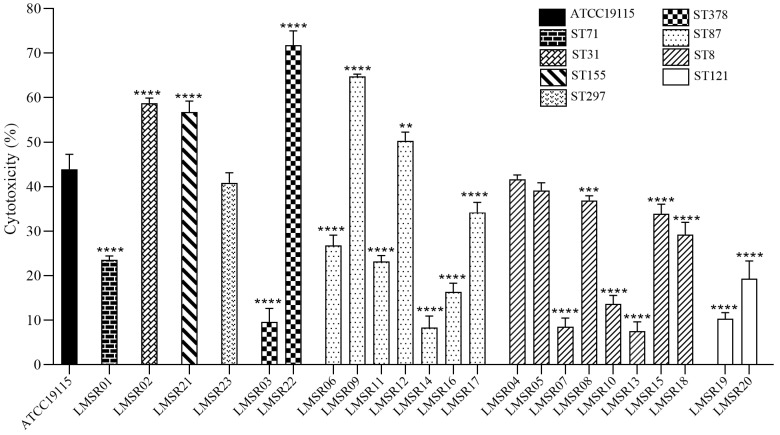
Comparative cytotoxicity of *L. monocytogenes* isolates in Caco-2 cells. ** *p* < 0.01, *** *p* < 0.001, **** *p* < 0.0001.

The cytotoxicity of 23 *L. monocytogenes* isolates was assessed using Caco-2 cells, revealing substantial variation in their toxic effects (Figure 1). The cytotoxicity of the isolates ranged from 7.6% to 71.8%. Among these, five isolates LMSR02, LMSR09, LMSR12, LMSR21 and LMSR22 exhibited significantly higher cytotoxicity than the positive control ATCC19115. In contrast, fifteen isolates showed significantly lower cytotoxicity than the positive control. Based on MLST results presented later, ST378 isolates demonstrated a broad cytotoxicity range (9.7–71.8%), whereas ST87 and ST8 isolates showed ranges of 8.4–64.8% and 7.6–41.7%, respectively. The cytotoxicity values of the ST71, ST31, ST155 and ST297 isolates (each from a single strain) were 23.6%, 58.8%, 56.8% and 40.9%, respectively. None of the isolates from ST8, ST71, ST121, or ST297 exceeded the cytotoxicity level of the positive control. Notably, ST121 isolates consistently exhibited lower cytotoxicity levels (10.4–19.3%) compared to ST387, ST87 and ST8 isolates. Among the five high cytotoxic isolates, LMSR12 was isolated from trout. The remaining four (LMSR02, LMSR09, LMSR21 and LMSR22) were isolated from salmon. Specifically, LMSR02 was obtained from a farmers’ market, while LMSR09, LMSR21 and LMSR22 were sourced from online stores.

#### 3.2.2. Mouse Survival Rate

**Figure 2:**
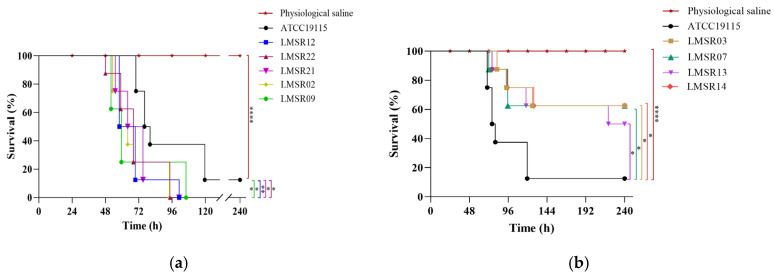
The survival rate of mice infected with five highly cytotoxic isolates (**a**) and four low cytotoxic isolates (**b**). * *p* < 0.05, ** *p* < 0.01, **** *p* < 0.0001.

To further confirm the virulence of five highly cytotoxic isolates (LMSR02, LMSR09, LMSR12, LMSR21, LMSR22), an in vivo assay was conducted using a mouse model to evaluate survival rates. Four low-cytotoxicity isolates (LMSR03, LMSR07, LMSR13, LMSR14) were also included in the assay. As shown in Figure 2, mice infected with the five highly cytotoxic isolates exhibited significantly lower survival rates compared to those in the ATCC 19115 positive control group. Similarly, mice infected with the four low-cytotoxicity isolates also showed significantly higher survival relative to the positive control. No mortality was observed in the blank control group throughout the experimental period. These results further validated the virulence levels of the isolates.

### 3.3. Antimicrobial Susceptibility of L. monocytogenes Isolates

The antimicrobial susceptibility profiles of 23 *L. monocytogenes* isolates are presented in Table 2. All isolates were resistant to CXM, CRO, FEP and NA, which is the same as the resistance profile observed in ATCC19115. Conversely, all isolates were sensitive to AMP, AMC, CN, K, TE, DO, FFC, IPM, MEM, VA and RD. Furthermore, ≥73.9% of isolates were susceptible to KZ, S, AK, CIP, OFX, C and F, whereas only 13% of isolates displayed resistant to SXT. It should be noted that the reference strain ATCC19115 showed susceptibility to all tested antibiotics except CXM, CRO, FEP and NA.

### 3.4. PCR-Serogroup of L. monocytogenes Isolates

The serogroup distribution of all isolates is presented in Table 3. Among the 23 isolates analyzed, three distinct serogroups were identified, including serogroup III (*n* = 1, 4.3%), I.1 (*n* = 15, 65.2%) and II.2 (*n* = 7, 30.4%).

### 3.5. WGS and Genetic Diversity of L. monocytogenes Isolates

#### 3.5.1. Genome Composition

**Figure 3:**
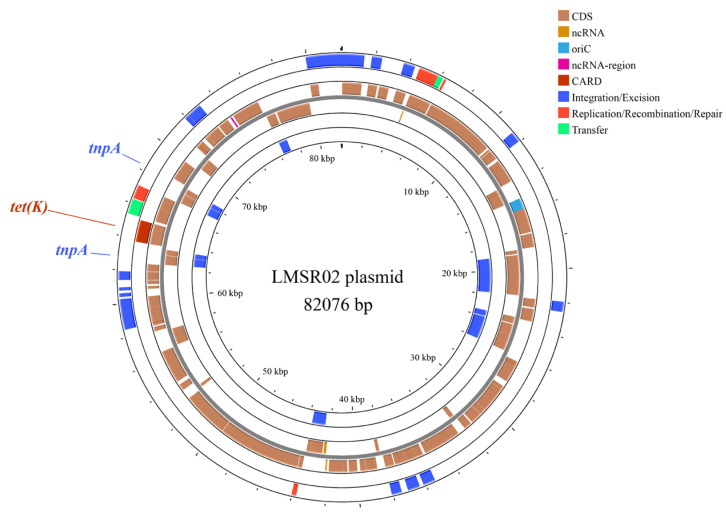
Circular genome map of the plasmid in the isolate LMSR02.

The genomic characteristics of 23 isolates are summarized in Table 4. The total genomic length of the isolates ranged from 2,816,405 bp to 3,207,701 bp, with the GC content between 37.95% and 39.12%, and the number of coding genes ranging from 2735 to 3169. Among the isolates, the number of tRNAs ranged from 52 to 67, and the number of rRNAs varied from 4 to 18. A plasmid was identified in the genome of LMSR02, and its map is presented in Figure 3. In addition, a total of 26 genomic islands and 15 prophages were predicted among the five highly cytotoxic isolates.

#### 3.5.2. MLST

Based on MLST analysis, the 23 *L. monocytogenes* isolates were originated from three distinct evolutionary lineages (lineage I, lineage II and lineage III), comprising eight STs that clustered into eight CCs (Table 3). The predominant STs identified were ST8 (CC8) and ST87 (CC87), accounting for eight and seven isolates, respectively. Additionally, two strains each were assigned to ST378 (CC19) and ST121 (CC121), while the remaining strains belonged to ST71 (CC131), ST31 (CC31), ST155 (CC155) and ST297 (CC7).

#### 3.5.3. Virulence Genes

**Figure 4:**
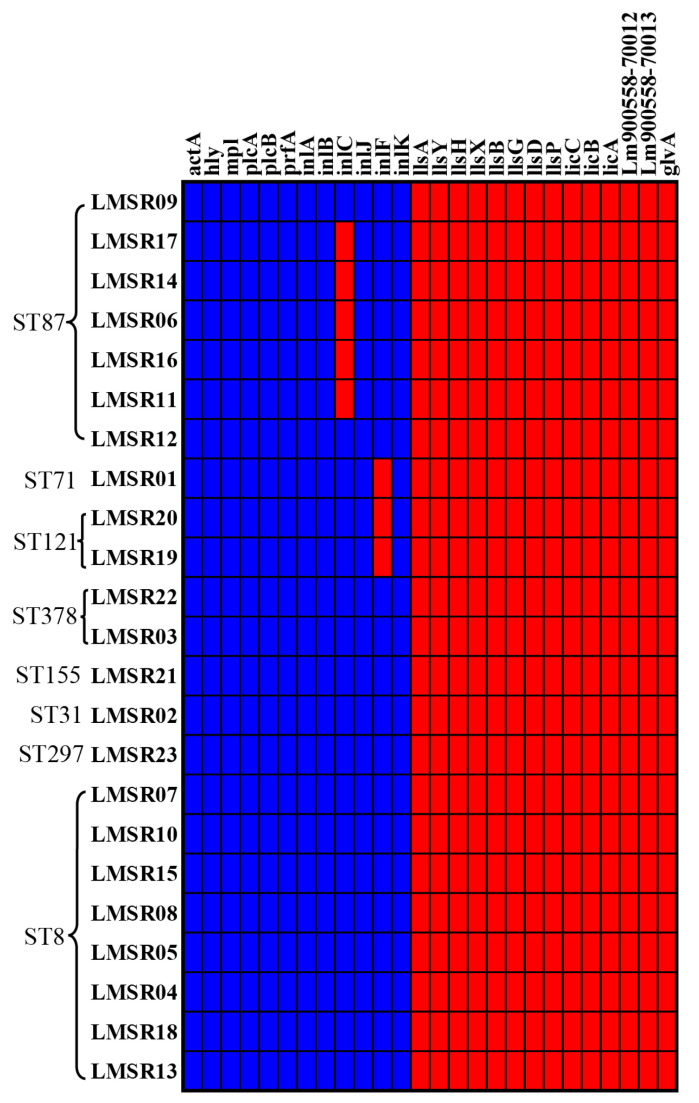
Heatmap showing the distribution of virulence genes of 23 *L. monocytogenes* isolates. Blue: presence of the gene, red: absence of the gene.

As illustrated in Figure 4, a heatmap displays the distribution of virulence genes across 23 *L. monocytogenes* isolates. In our study, all isolates carried the full complement of LIPI-1-associated virulence genes (*actA*, *hly*, *mpl*, *plcA*, *prfA*, and *plcB*). Additionally, the majority of isolates contained six internalin genes (*inlA*, *inlB*, *inlC*, *inlF*, *inlJ* and *inlK*), with the exception of ST87 isolates, which lacked *inlC*, and ST71 and ST121 isolates, which lacked *inlF*. The virulence genes associated with LIPI-3 (*llsA*, *llsP*, *llsB*, *llsH*, *llsX*, *llsY*, *llsD* and *llsG*) and LIPI-4 (*Lm900558-70012*, *Lm900558-70013*, *licC*, *licB*, *licA* and *glvA*) were absent in all isolates.

#### 3.5.4. Antimicrobial Resistance Genes

The presence of antimicrobial resistance genes and their associated mechanisms of 23 *L. monocytogenes* isolates are summarized in Table 5. All isolates harbored genes conferring resistance to lincosamides (*lin*) and phosphonic acid (*FosX*), both of which function through enzymatic inactivation. Other resistance genes, including *msr(G)*, *mef(F)*, *fexA*, *norB* and *qacJ*, were detected in certain isolates. Additionally, the tetracycline resistance gene *tet(K)*, which was present in LMSR02, was located within a transposon on a plasmid (Figure 3). This configuration suggested that the resistance gene could be transmitted to other strains via transposition, thereby increasing the risk of resistance dissemination. Overall, the antimicrobial resistance gene profiles of all isolates in this study were similar. However, there was poor consistency between the phenotypic resistance patterns and the genotypic predictions, suggesting potential regulatory effects at the post-transcriptional or post-translational levels, or the presence of undetected resistance mechanisms.

#### 3.5.5. SNP-Based Evolutionary Relationship

**Figure 5:**
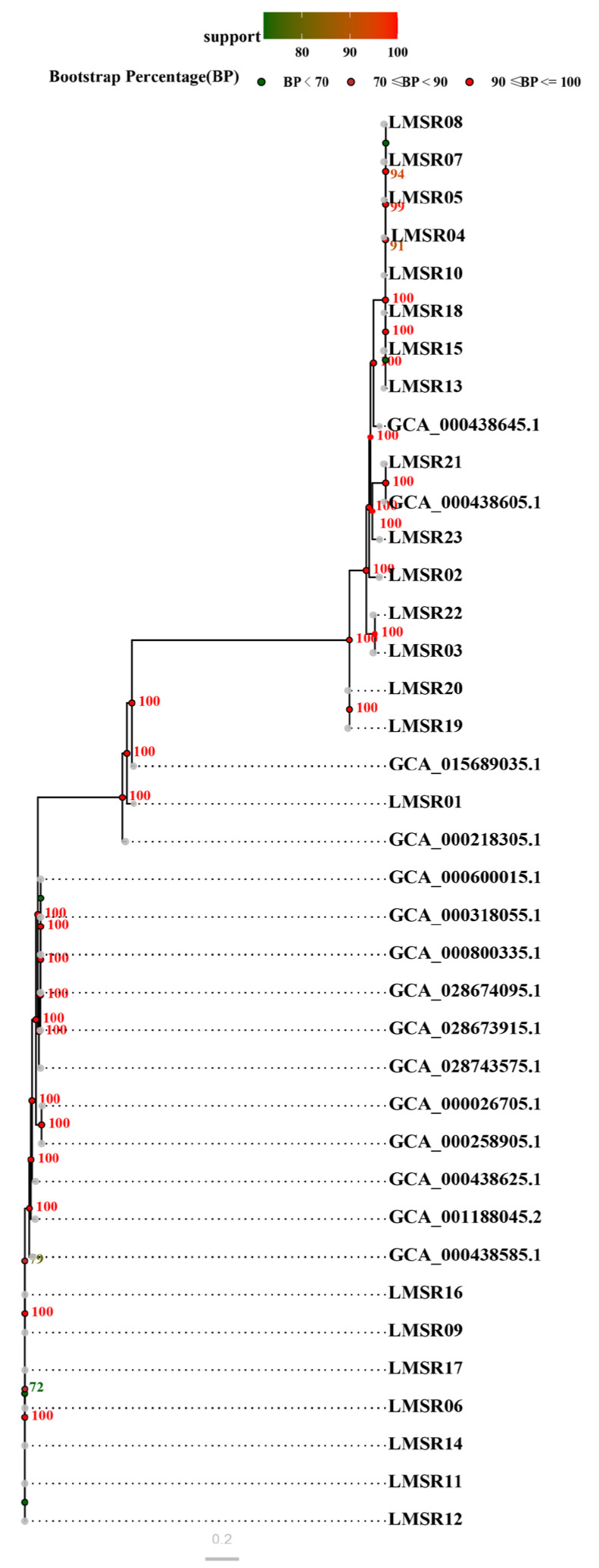
SNP-based phylogenetic tree of 38 *L. monocytogenes* isolates.

The SNP analysis of 38 isolates was performed, as shown in Figure 5. The resulting SNP tree was divided into four principal branches, suggesting that these strains originated from four distinct evolutionary lineages. The 23 isolates sharing the same CC were consistently grouped together within these clusters.

#### 3.5.6. ANI and Collinearity

**Figure 6:**
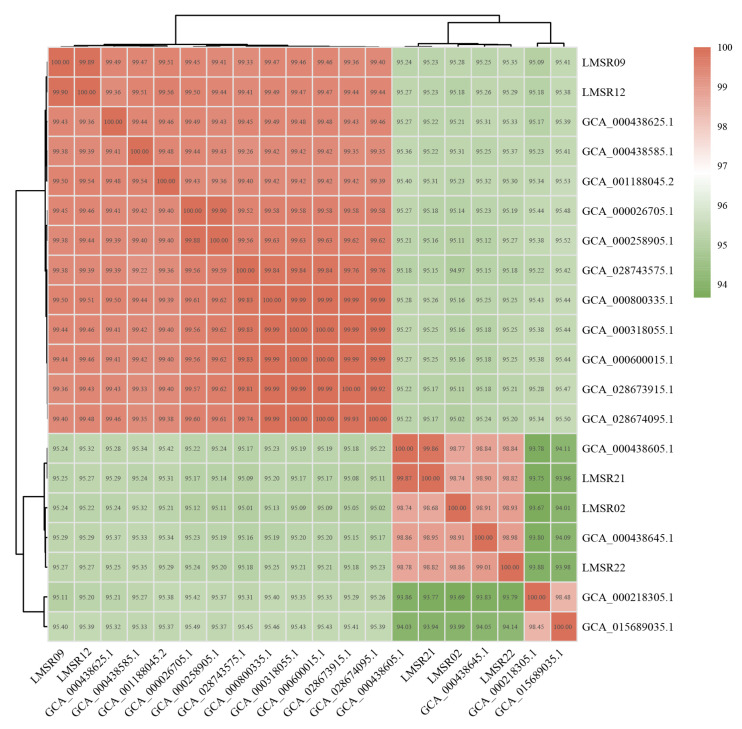
Heatmap of ANI analysis of 20 *L. monocytogenes* isolates.

**Figure 7:**
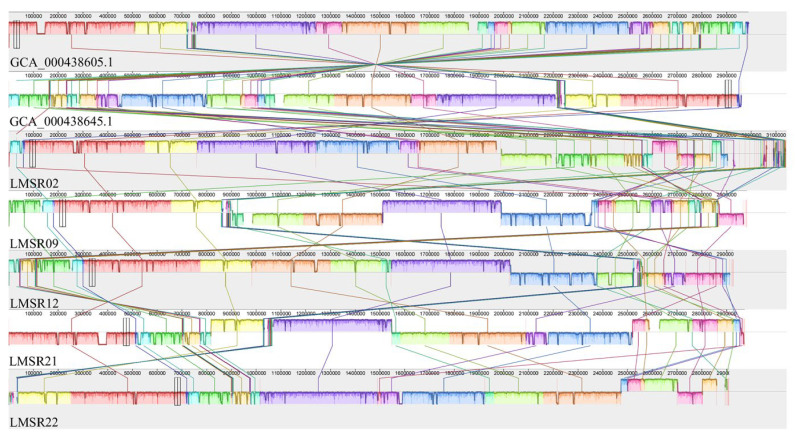
Collinearity analysis of whole genomes of 7 *L. monocytogenes* isolates.

The ANI results for five highly cytotoxic isolates and 15 *L. monocytogenes* isolates obtained from NCBI are presented in Figure 6. The results indicated that five highly cytotoxic isolates have the closest genetic relationship with J2-031 (GCA_000438645.1) and C1-387 (GCA_000438605.1). Comparative genomics (Figure 7) indicated a close evolutionary relationship between five highly cytotoxic isolates and two other stains J2-031 (GCA_000438645.1) and C1-387 (GCA_000438605.1), with strong genomic synteny among all seven strains. The genomic module compositions of the seven strains were similar, but the specific positions and arrangement sequences of these modules in the genome were different. In some strains, gaps were present within the same collinear modules. These vacant regions are likely to represent strain-specific sequences, which may correspond to genomic islands, prophages, or unique insertion sequences acquired through horizontal gene transfer.

## 4. Discussion

Fish is commonly contaminated with *L. monocytogenes* through bacterial dissemination from intestinal contents to muscle tissues and/or cross-contamination resulting from the unhygienic handling and processing environment [33]. Regardless of the contamination route, consumers remain at risk of *L. monocytogenes* infection from retail fish products. Therefore, it is necessary to investigate the contamination levels of *L. monocytogenes* in retail raw salmon and trout products. In this study, *L. monocytogenes* was detected in 15.3% of retail raw salmon and trout products with contamination levels ranging from 0.36 to 1100 MPN/g. There are few studies that analyze the quantitative contamination levels of *L. monocytogenes* in raw salmon and trout products, making direct comparisons challenging. However, the occurrence of *L. monocytogenes* in our study was lower than those reported by Simonaviciene et al. [34] and Mahgoub et al. [35] but higher than the findings of Zhang et al. [36] and Leong et al. [37]. The contamination rates of *L. monocytogenes* varied depending on the retail sources. In the samples collected from physical stores, no *L. monocytogenes* was detected in samples from specialty stores, while the highest detection rate of *L. monocytogenes* was found in samples from farmers’ markets. Notably, samples from e-commerce platforms exhibited the highest prevalence (30%), including the most heavily contaminated sample (1100 MPN/g). In China, the national standard GB 29921-2021 specifies a maximum permissible level of *L. monocytogenes* in ready-to-eat raw animal aquatic products at 100 CFU/g. In the present study, although only one sample exceeded this limit, several samples tested positive, which may pose a risk to consumers. These findings also suggested that consumers may reduce their risk by purchasing raw salmon and trout from exclusive stores, which typically maintain higher hygiene and safety standards. The improvement of facility environments can make them less conducive to the growth of *L. monocytogenes* [38]; therefore, to enhance the safety of raw salmon and trout products, corresponding measures should be taken to improve the sanitation in aquaculture waters and processing environment.

*L. monocytogenes* has been classified into 13 serotypes based on O and H antigen profiling [1]. Among these, serotypes 1/2a and 1/2b are frequently isolated from food sources [26,39,40], with 1/2a being the predominant serotype detected in Chinese food products [41]. In this study, 65.2% of the isolates belonged to serogroup I.1 (1/2a, 3a) and 30.4% of the isolates belonged to serogroup II.2 (1/2b, 3b, 7), which is in agreement with the results of previous reports [42,43,44]. Furthermore, Zhang et al. [40] observed that strains belonging to the same CC shared the same serotype, which aligns with our study. *L. monocytogenes* strains can be phylogenetically categorized into four evolutionary lineages (Ⅰ, II, III and IV), with lineage II being the most prevalent among isolates from foods and environmental sources [45]. As previously reported, there were 65.2% of *L. monocytogenes* isolates that belonged to evolutionary lineage II. Among the eight CCs identified (CC87, CC8, CC19, CC121, CC155, CC7, CC31 and CC131), seven have been associated with clinical cases both domestically and internationally [46,47]. Notably, CC155 isolates, which accounted for only 4.3% of the raw salmon and trout products analyzed here, have been frequently detected in food processing environments in the United States and Canada [48]. Similarly, CC7 strains, represented by a single isolate in this study, were responsible for 23% listeriosis cases in Norway [47]. Previous studies have reported that ST8, ST87 and ST121 strains are commonly found in foodborne isolates [39,40]. Our findings further support this observation, with ST8 (34.8%), ST87 (30.4%) and ST121 (8.7%) being the dominant STs in raw salmon and trout products. However, it is important to note that all *L. monocytogenes* STs are pathogenic and pose equivalent food safety risks [21]. Notably, ST5, ST3, ST8 and ST87 strains have been closely related to listeriosis [46,49]. In the present study, all ST8 and ST87 *L. monocytogenes* strains were from raw salmon and trout products purchased from e-commerce platforms, underscoring the need for strengthening the monitoring of online retailers selling these products to mitigate potential public health risks.

*L. monocytogenes* is an important foodborne pathogen with considerable potential virulence, which could pose a serious threat to consumers’ health. To assess its pathogenic risk, we evaluated the cytotoxicity of *L. monocytogenes* isolates and employed WGS to characterize their virulence gene profiles. Consistent with previous reports, ST121 strains, known as low-virulence strains within evolutionary lineage II [19,50], exhibited lower cytotoxicity in our study. In contrast, ST87 strains, recognized as hypervirulent in China [51], displayed different levels of cytotoxicity in our study. The pathogenicity of *L. monocytogenes* is closely related to its virulence genes and pathogenicity islands [52,53]. The mechanism is the result of numerous virulence factors under the complex regulation of various regulatory factors. Therefore, studying the virulence genes of *L. monocytogenes* is helpful for understanding its pathogenic mechanism. LIPI-1 is composed of six genes (*prfA*, *actA*, *plcA*, *plcB*, *hly* and *mpl*) and universally present in all *L. monocytogenes* [19]. The virulence genes associated with LIPI-1 and internalin genes are responsible for the intracellular and intercellular motility of *L. monocytogenes* and play an essential role in its pathogenic and virulence [54,55]. As expected, all isolates in our study carried LIPI-1 and multiple internalin genes (*inlA*, *inlB*, *inlJ* and *inlK*). However, the absence of *inlC* and *inlF* were observed in several isolates, which was consistent with other reports [56]. Listeriolysin S (LLS) encoded by LIPI-3 (*llsA*, *llsP*, *llsB*, *llsH*, *llsX*, *llsY*, *llsD* and *llsG*) is a cytotoxic factor [52]. In our study, LIPI-3 was not detected. LIPI-4, associated with neural and placental infection [51], is typically linked to hypervirulent strains [57]. Although LIPI-4 is commonly found in CC4 strains [58], none was detected in this study. Interestingly, isolates belonging to the same CC and carrying the same virulence gene exhibited different virulence levels, which is consistent with the finding reported by Wu et al. [43]. Consequently, additional virulence mechanisms have yet to be identified and characterized.

In general, *L. monocytogenes* strains are sensitive to most antibiotics [59]. The current first-line treatment for listeriosis consists of a combination of AMP or penicillin with CN [1], while SXT is considered as a secondary option for therapy [59]. In addition, TE and VN have also been employed in the treatment of listeriosis [1]. Fortunately, all *L. monocytogenes* isolated in this study exhibited sensitivity to AMP, CN, TE and VA. All isolates were resistant to cephalosporins (CXM, CRO and FEP) and nalidixic acid, which is due to intrinsic resistance of the strain to these drugs [60]. These drugs are not recommended for inclusion as monitoring subjects in future surveillance. Out of the tested isolates, three isolates were resistant to SXT. Although the antimicrobial resistance of these isolates from raw salmon and trout products is not that severe, enhanced surveillance is still necessary. Our study found that there was a discrepancy between the antimicrobial resistance phenotype and genotype. All *L. monocytogenes* isolates were resistant to CXM, CRO, FEP and NA; however, no corresponding resistance genes were detected. This suggests that non-specific efflux mechanisms may contribute to their resistance. This hypothesis needs to be confirmed through future in-depth research. Furthermore, strain LMSR02, which harbored the *tet(K)* gene within a transposon on its plasmid, exhibited phenotypic sensitivity to TE. This may be attributed to the lack of expression of the antimicrobial resistance gene under normal conditions; however, upon exposure to external stimuli, expression may be induced. Moreover, the genetic context suggests that the resistance gene could be transferred to other strains via transposition, thereby increasing the potential for dissemination of resistance. Given the limitations of phenotypic susceptibility testing in assessing a broad spectrum of antimicrobials, WGS has emerged as a powerful tool for comprehensive resistance gene identification. WGS not only compensates for the inadequacy of antimicrobial susceptibility test but also facilitates the prediction of the evolution of antimicrobial resistance of bacteria, thereby supporting the development of new antimicrobials.

WGS has emerged as a powerful tool for elucidating comprehensive genomic characteristics and evolutionary potential of bacterial strains, thereby facilitating systematic screening for genetic elements associated with adaptive evolution. In the context of *L*. *monocytogenes*, a pathogen of significant public health concern, WGS enables high-resolution analysis of genomic architecture and functional plasticity. Genomic islands and prophages are known to mediate horizontal gene transfer and serve as crucial vehicles for bacteria to acquire exogenous genetic material, such as virulence genes, antimicrobial resistance genes and other adaptive determinants [61]. A total of 26 genomic islands and 15 prophages were predicted in the genomes of LMSR02, LMSR09, LMSR12, LMSR21 and LMSR22, indicating that *L. monocytogenes* isolates are highly likely to employ such mobile genetic elements for self-protection and enhanced environmental adaptability. The ANI analysis of 20 *L. monocytogenes* strains revealed that J2-031 (GCA_000438645.1) and C1-387 (GCA_000438605.1) had the closest genetic relationship with the five isolates. This close relatedness suggests possible common ancestry or shared ecological niches among these strains. The collinearity analysis of seven isolates showed that the genomic module compositions of the seven strains were similar, yet marked rearrangements and inversions were observed among them. This suggested that *L. monocytogenes*, while maintaining the genomic stability of its core genes, may enhance its adaptability and pathogenic potential in specific environments through local rearrangement and inversion. Such structural variations could modulate gene expression, alter regulatory networks, or facilitate the acquisition of novel traits, thereby promoting survival under selective pressures. Furthermore, tracing the origins of the strains provides compelling evidence for contamination source tracking. The SNP-based phylogenetic tree of the 23 *L. monocytogenes* isolates revealed that strains isolated from Japanese restaurants, farmers markets, and e-commerce platforms clustered within a third major branch. This clustering pattern suggests the potential existence of undiscovered common transmission chains or shared food distribution networks, highlighting the possible circulation of related strains across diverse retail and food service environments. These findings underscore the value of genomic surveillance in understanding the dissemination routes of *L. monocytogenes* and in supporting targeted interventions for food safety management.

## 5. Conclusions

This study revealed the prevalence, characterization and genetic diversity of *L*. *monocytogenes* in ready-to-eat raw salmon and trout products collected from retail markets in Qingdao, China, and online stores located in different provinces with top 10 sales volume on e-commerce platforms. The occurrence of *L. monocytogenes* in 150 samples was 15.3%, with the contamination level ranging from 0.36 to 1100 MPN/g. These isolates carried similar antimicrobial resistance genes and virulence genes, exhibited sensitivity to most of the tested antibiotics and showed varying degrees of cytotoxicity. Five of these isolates were highly virulent compared with reference strain *L*. *monocytogenes* ATCC 19115. Three serogroups were identified in these isolates, with serogroup I.1 (1/2a, 3a) being the predominant one. All isolates were from three evolutionary lineages belonging to eight STs. The most prevalent STs were the ST8 and ST87 strains, both of which are strongly associated with human listeriosis cases. Five highly virulent isolates have the closest genetic relationship with J2-031 (GCA_000438645.1) and C1-387 (GCA_000438605.1). This finding suggests that raw salmon-and trout products may serve as a vector for *L*. *monocytogenes* transmission, thereby constituting a significant public health risk. The results of this study offer valuable insights for regulatory authorities in China to formulate effective strategies for the prevention and control of *L. monocytogenes* contamination. Moreover, these findings contribute to improving the food safety management of ready-to-eat raw salmon and trout products at the retail level, thereby better protecting consumer health.

## Figures and Tables

**Table 1:** Contamination levels of *L. monocytogenes* in retail raw salmon and trout products.

Sample Source	No (%) of Positive Samples	Contamination Level (MPN/g)
Exclusive store	0% (0/15)	<0.3
Large supermarkets	2.9% (1/35)	0.36
Japanese restaurants	12% (3/25)	2.3~43
Farmers markets	16% (4/25)	0.92~9.2
E-commerce platforms	30% (15/50)	0.92~1100
Total	15.3% (23/150)	<0.3~1100

**Table 2:** Antimicrobial susceptibility of 23 *L. monocytogenes* isolates.

Antimicrobials	ATCC19115	No. (%) of Isolates		
		R	I	S
AMP	S	0 (0%)	0 (0%)	23 (100%)
AMC	S	0 (0%)	0 (0%)	23 (100%)
KZ	S	0 (0%)	3 (13%)	20 (87%)
CXM	R	23 (100%)	0 (0%)	0 (0%)
CRO	R	23 (100%)	0 (0%)	0 (0%)
FEP	R	23 (100%)	0 (0%)	0 (0%)
S	S	0 (0%)	3 (13%)	20 (87%)
AK	S	0 (0%)	1 (4.3%)	22 (95.7%)
CN	S	0 (0%)	0 (0%)	23 (100%)
K	S	0 (0%)	0 (0%)	23 (100%)
NA	R	23 (100%)	0 (0%)	0 (0%)
CIP	S	0 (0%)	5 (21.7%)	18 (78.3%)
OFX	S	0 (0%)	6 (26.1%)	17 (73.9%)
TE	S	0 (0%)	0 (0%)	23 (100%)
DO	S	0 (0%)	0 (0%)	23 (100%)
SXT	S	3 (13%)	9 (39.1%)	11 (47.8%)
C	S	0 (0%)	3 (13%)	20 (87%)
FFC	S	0 (0%)	0 (0%)	23 (100%)
F	S	0 (0%)	3 (13%)	20 (87%)
IPM	S	0 (0%)	0 (0%)	23 (100%)
MEM	S	0 (0%)	0 (0%)	23 (100%)
VA	S	0 (0%)	0 (0%)	23 (100%)
RD	S	0 (0%)	0 (0%)	23 (100%)

Notes: S: susceptible, I: intermediate, R: resistant, AMP: ampicillin, AMC: amoxycillin, KZ: cefazolin, CXM: cefuroxime sodium, CRO: ceftriaxone, FEP: cefepime, IPM: imipenem, MEM: meropenem, S: streptomycin, AK: amikacin, CN: gentamicin, K: kanamycin, NA: nalidixicacid, CIP: ciprofloxacin, OFX: ofloxacin, TE: tetracycline, DO: doxycycline, SXT: sulphamethoxazole-trimethoprim, C: chloramphenicol, FFC: florfenicol, F: nitrofurantoin, VA: vancomycin, RD: rifampicin.

**Table 3:** Molecular serogroup and MLST of 23 *L. monocytogenes* isolates.

Isolate	ST	CC	Evolutionary Lineage	Records of Seven Housekeeping Genes							Molecular Serogroup
				*abcZ*	*bglA*	*cat*	*dapE*	*dat*	*ldh*	*lhkA*	
LMSR01	71	CC131	III	18	11	21	24	17	35	13	III
LMSR02	31	CC31	II	7	14	10	19	9	8	1	I.1
LMSR03	378	CC19	II	7	49	19	6	1	24	1	I.1
LMSR04	8	CC8	II	5	6	2	9	5	3	1	I.1
LMSR05	8	CC8	III	5	6	2	9	5	3	1	I.1
LMSR06	87	CC87	Ⅰ	12	1	4	14	3	39	4	II.2
LMSR07	8	CC8	II	5	6	2	9	5	3	1	I.1
LMSR08	8	CC8	II	5	6	2	9	5	3	1	I.1
LMSR09	87	CC87	Ⅰ	12	1	4	14	3	39	4	II.2
LMSR10	8	CC8	II	5	6	2	9	5	3	1	I.1
LMSR11	87	CC87	Ⅰ	12	1	4	14	3	39	4	II.2
LMSR12	87	CC87	I	12	1	4	14	3	39	4	II.2
LMSR13	8	CC8	II	5	6	2	9	5	3	1	I.1
LMSR14	87	CC87	I	12	1	4	14	3	39	4	II.2
LMSR15	8	CC8	II	5	6	2	9	5	3	1	I.1
LMSR16	87	CC87	I	12	1	4	14	3	39	4	II.2
LMSR17	87	CC87	I	12	1	4	14	3	39	4	II.2
LMSR18	8	CC8	II	5	6	2	9	5	3	1	I.1
LMSR19	121	CC121	II	7	6	8	8	6	37	1	I.1
LMSR20	121	CC121	II	7	6	8	8	6	37	1	I.1
LMSR21	155	CC155	II	7	10	16	7	5	2	1	I.1
LMSR22	378	CC19	II	7	49	19	6	1	24	1	I.1
LMSR23	297	CC7	II	5	42	5	7	6	2	1	I.1

Notes: ST: sequence type, CC: clonal complex.

**Table 4:** The genome features of 23 *L. monocytogenes* isolates.

Isolate	Genome Length (bp)	GC Content (%)	Coding Genes	tRNAs	rRNAs	Genome Island	Prophage
LMSR01	2,816,405	38.95	2735	57	6	/	/
LMSR02	3,207,701	38.00	3169	67	18	9	6
LMSR03	2,910,141	39.00	2862	54	6	/	/
LMSR04	2,950,252	39.04	2920	55	5	/	/
LMSR05	2,948,969	39.12	2920	52	4	/	/
LMSR06	2,982,671	39.00	2943	54	6	/	/
LMSR07	2,924,089	38.85	2899	56	5	/	/
LMSR08	2,949,838	38.77	2920	55	5	/	/
LMSR09	3,009,347	38.00	2916	67	18	4	2
LMSR10	2,950,695	38.08	2925	55	5	/	/
LMSR11	2,928,348	38.14	2856	54	6	/	/
LMSR12	2,954,949	38.00	2839	67	18	2	1
LMSR13	2,950,749	38.08	2924	55	5	/	/
LMSR14	2,984,867	38.32	2945	54	6	/	/
LMSR15	2,949,344	38.51	2922	53	4	/	/
LMSR16	2,969,272	38.19	2931	54	5	/	/
LMSR17	2,981,308	38.25	2939	56	6	/	/
LMSR18	2,950,431	37.95	2924	53	4	/	/
LMSR19	3,140,506	38.20	3150	54	5	/	/
LMSR20	3,094,697	38.40	3075	54	5	/	/
LMSR21	3,000,885	38.00	2934	67	18	7	3
LMSR22	2,941,341	38.00	2870	67	18	4	3
LMSR23	2,996,143	38.30	2998	54	7	/	/

Note: “/” means not applicable.

**Table 5:** Antimicrobial resistance genes and mechanisms of 23 *L. monocytogenes* isolates.

Resistance Mechanism	Antimicrobial Resistance Genes	Antibiotic Class	L M S R 01	L M S R 02	L M S R 03	L M S R 04	L M S R 05	L M S R 06	L M S R 07	L M S R 08	L M S R 09	L M S R 10	L M S R 11	L M S R 12	L M S R 13	L M S R 14	L M S R 15	L M S R 16	L M S R 17	L M S R 18	L M S R 19	L M S R 20	L M S R 21	L M S R 22	L M S R 23
target protection	*msr(G)*	macrolide streptogramin	–	+	–	–	–	–	–	–	–	–	–	–	–	–	–	–	–	–	–	–	–	–	–
efflux	*tet(K)*	tetracycline	–	+	–	–	–	–	–	–	–	–	–	–	–	–	–	–	–	–	–	–	–	–	–
	*mef(F)*	macrolide	–	+	–	–	–	–	–	–	–	–	–	–	–	–	–	–	–	–	–	–	–	–	–
	*fexA*	phenicol	–	+	–	–	–	–	–	–	–	–	–	–	–	–	–	–	–	–	–	–	–	–	–
	*norB*	fluoroquinolone	–	+	–	–	–	–	–	–	–	–	–	–	–	–	–	–	–	–	+	+	–	–	+
	*qacJ*	disinfecting agents and antiseptics	–	–	–	–	–	–	–	–	–	–	–	–	–	–	–	–	–	–	+	+	–	–	–
inactivation	*lin*	lincosamide	+	+	+	+	+	+	+	+	+	+	+	+	+	+	+	+	+	+	+	+	+	+	+
	*FosX*	phosphonic acid	+	+	+	+	+	+	+	+	+	+	+	+	+	+	+	+	+	+	+	+	+	+	+

Note: “+” represents presence, “–” represents absence.

## Data Availability

The sequences obtained in this study have been deposited in the NCBI database under the accession numbers corresponding to their respective strain numbers as follows: JBTMWJ000000000, JBTNQN000000000, JBTMWI000000000, JBTMWH000000000, JBTMWG000000000, JBTMWF000000000, JBTMWE000000000, JBTMWD000000000, JBTNQO000000000, JBTMWC000000000, JBTMWB000000000, JBTNQP000000000, JBTMWA000000000, JBTMVZ000000000, JBTMVY000000000, JBTMVX000000000, JBTMVW000000000, JBTMVV000000000, JBTMVU000000000, JBTMVT000000000, JBTNQR000000000, JBTNQQ000000000, JBTMVS000000000. All reference genomes used in this study were obtained from publicly accessible resources on NCBI and are not subject to any reuse restrictions.
